# Bromide of Potassium to Prevent Nausea Following the Use of Opium

**Published:** 1871-03

**Authors:** J. Y. Dale

**Affiliations:** Lemont, Pa.


### BROMIDE OF POTASSIUM TO PREVENT THE NAUSEA FOLLOWING THE USE OF OPIUM.

BY J. Y. DALE, M. D., OF LEMONT, PA.

In May, 1870, I was called to see an old lady who was suffering with lumbago. The pain was excruciating, and the least attempt at movement caused intense agony. Not having my hypodermic syringe with me, I gave her the third of a grain of morphia by the stomach. She recognized the medicine by its taste, and said that the sickness it caused was almost as bad as the pain; for on several previous occasions she had taken morphia, and each time the nausea was very severe, and continued for a week. I prescribed smaller doses to be given at intervals during the night, if necessary, in connection with other treatment, and on my return next morning, found the pain much mitigated. She begged for something to prevent the nausea, and I could think of nothing that would be so likely to have this effect as bromide of potassium; so by way of testing its virtues in this respect, I prescribed ten grains to be taken every three hours, with directions to continue the morphia in one-eighth of a grain doses if the pain were severe enough to require it. Convalesence was rapid; in two days my patient was able to walk, and not a single unpleasant symptom manifested itself. I have since used the bromide where there was the same idiosyncrasy in regard to opium, with similar good results.

